# Validity Evidence of the eHealth Literacy Questionnaire (eHLQ) Part 2: Mixed Methods Approach to Evaluate Test Content, Response Process, and Internal Structure in the Australian Community Health Setting

**DOI:** 10.2196/32777

**Published:** 2022-03-08

**Authors:** Christina Cheng, Gerald R Elsworth, Richard H Osborne

**Affiliations:** 1 School of Health Sciences Centre for Global Health and Equity Swinburne University of Technology Hawthorn Australia; 2 Faculty of Health School of Health and Social Development Deakin University Burwood Australia

**Keywords:** eHealth, health literacy, health equity, questionnaire design, validity evidence, eHLQ, mobile phone

## Abstract

**Background:**

Digital technologies have changed how we manage our health, and eHealth literacy is needed to engage with health technologies. Any eHealth strategy would be ineffective if users’ eHealth literacy needs are not addressed. A robust measure of eHealth literacy is essential for understanding these needs. On the basis of the eHealth Literacy Framework, which identified 7 dimensions of eHealth literacy, the eHealth Literacy Questionnaire (eHLQ) was developed. The tool has demonstrated robust psychometric properties in the Danish setting, but validity testing should be an ongoing and accumulative process.

**Objective:**

This study aims to evaluate validity evidence based on test content, response process, and internal structure of the eHLQ in the Australian community health setting.

**Methods:**

A mixed methods approach was used with cognitive interviewing conducted to examine evidence on test content and response process, whereas a cross-sectional survey was undertaken for evidence on internal structure. Data were collected at 3 diverse community health sites in Victoria, Australia. Psychometric testing included both the classical test theory and item response theory approaches. Methods included Bayesian structural equation modeling for confirmatory factor analysis, internal consistency and test-retest for reliability, and the Bayesian multiple-indicators, multiple-causes model for testing of differential item functioning.

**Results:**

Cognitive interviewing identified only 1 confusing term, which was clarified. All items were easy to read and understood as intended. A total of 525 questionnaires were included for psychometric analysis. All scales were homogenous with composite scale reliability ranging from 0.73 to 0.90. The intraclass correlation coefficient for test-retest reliability for the 7 scales ranged from 0.72 to 0.95. A 7-factor Bayesian structural equation modeling using small variance priors for cross-loadings and residual covariances was fitted to the data, and the model of interest produced a satisfactory fit (posterior productive *P*=.49, 95% CI for the difference between observed and replicated chi-square values −101.40 to 108.83, prior-posterior productive *P*=.92). All items loaded on the relevant factor, with loadings ranging from 0.36 to 0.94. No significant cross-loading was found. There was no evidence of differential item functioning for administration format, site area, and health setting. However, discriminant validity was not well established for scales 1, 3, 5, 6, and 7. Item response theory analysis found that all items provided precise information at different trait levels, except for 1 item. All items demonstrated different sensitivity to different trait levels and represented a range of difficulty levels.

**Conclusions:**

The evidence suggests that the eHLQ is a tool with robust psychometric properties and further investigation of discriminant validity is recommended. It is ready to be used to identify eHealth literacy strengths and challenges and assist the development of digital health interventions to ensure that people with limited digital access and skills are not left behind.

## Introduction

### Background

Digital technologies have brought fundamental changes to modern-day life including how we manage our health. We can quickly search for health information at our fingertips but are also facing an avalanche of misinformation, as evident during the COVID-19 pandemic [1]. We can have instant access to our electronic personal health record, but these digital systems can be difficult to use or do not meet our expectation [2-5]. In addition, some people simply do not have or only have limited access or skills to use technologies for health, leading to the potential widening of health inequity when people with limited access or skills are being left behind in the digital age.

To characterize the challenges of accessing and using digital technologies for health, the concept of eHealth literacy was coined in 2006 [6]. At that time, it was defined as “the ability to seek, find, understand, and appraise health information from electronic sources and apply the knowledge gained to addressing or solving a health problem” [6]. It is further recognized that any digital or eHealth strategy or intervention would be ineffective if the eHealth literacy needs of users are not addressed [6-8]. For example, in the postevaluation phase of the now defunct web-based personal health record in the United Kingdom, the HealthSpace, it was concluded that there was a mismatch of the system and users’ expectations, and some users seemed to lack the health literacy and digital literacy required to use the system [2,3].

### The eHealth Literacy Questionnaire

To describe and address eHealth literacy needs, Norgaard et al [9] developed the eHealth Literacy Framework using a grounded validity-driven approach based on the results of a series of concept mapping workshops with a diverse range of stakeholders. Therefore, 7 dimensions of eHealth literacy were identified, depicting an overarching vision of how people access, understand, and use technology for health involving skills, health systems, and interaction between the individual and the systems [9]. This grounded framework provides a more comprehensive and contemporary view of eHealth literacy than the original definition, as it also taps into the role of digital systems and the interaction between users and systems. The framework was subsequently used to develop the eHealth Literacy Questionnaire (eHLQ) as a tool to measure eHealth literacy based on the seven dimensions:

1. Using technology to process health information
2. Understanding of health concepts and language
3. Ability to actively engage with digital services
4. Feel safe and in control
5. Motivated to engage with digital services
6. Access to digital services that work
7. Digital services that suit individual needs [10]

With the inclusion of eHealth literacy dimensions relating to user interaction and experiences in using digital health systems, the eHLQ embraces the real-world experiences of users while capturing the interactivity and increasing capabilities of digital technologies. It can provide rich information about the competencies of individuals as well as the maturity of digital health systems, as mature systems are likely to be more responsive to the individual needs of users [10]. As such, the eHLQ is a useful tool for digital health developers and implementers to promote equity-driven digital health systems and eventually contribute to health equity.

The eHLQ was simultaneously developed in Danish and English to avoid idiom and improve item wording to enhance future translation of the questionnaire into other languages [10]. The tool consists of 35 items with 7 scales, each with 4 to 6 items, and the response option is a 4-point ordinal scale of *strongly disagree* to *strongly agree*. The results are 7 equally weighted composite scale scores [10]. Initial validity testing of the Danish version of the eHLQ, based on data collected from 475 people from community and health care settings in Denmark, demonstrated the psychometric robustness of the tool. A 7-factor confirmatory factor analysis (CFA) model using the Bayesian structural equation modeling (BSEM) approach resulted in a satisfactory fit. All the 35 items of the tool loaded strongly on their relevant factors (range 0.36-0.94) with no significant cross-loadings. Good internal consistency was also demonstrated with satisfactory composite scale reliability for each of the 7 scales (range 0.75-0.87). In addition to taking the classical test theory (CTT) approach to the testing of psychometric properties, the item response theory (IRT) approach was also used. The results confirmed that the 35 items represented a range of difficulties and had good discrimination for testing people with different levels of eHealth literacy ability. Measurement invariance for age and sex was also demonstrated [10]. Although the study provided satisfactory validity evidence of the eHLQ in the Danish setting, evidence for the English version needs to be established. Described as a pioneer of eHealth in the world, Denmark has a national, publicly owned eHealth portal that is used by at least 2.3 million unique users out of their 5.8 million citizens per month as of 2019 [11,12]. Digital health is part of the routine for many Danish citizens. Although Australia also has a national digital health record system, information from an Australian Senate estimates hearing in 2019 revealed that only 4% of Australians had logged in to the Australian system [13]. Therefore, how the eHLQ will perform in settings with less prominent public use of digital health services is not known.

### Validity Evidence

Validity testing is an ongoing process that involves the accumulation of 5 sources of evidence based on test content, response process, internal structure, relations to other variables, and consequences of testing, according to the authoritative reference of developing and using of educational and psychological measurements, the *Standards for Educational and Psychological Testing* (the *Standards*) [14].

Evidence based on test content is used to determine whether the items represent the content domain and may also include whether the wordings are easy to read and formats of administration are easy to use. Response process refers to the cognitive process of survey participants, that is, whether the interpretation of the items by participants aligns with the intended interpretation of items by test developers. It may also include whether interpretation remains the same across subgroups or across different formats of administration. Internal structure is the extent to which items conform to the constructs and relates to aspects such as factor analysis, reliability, and measurement invariance. Relations to other variables is the analysis of the relationship between the scores on another instrument relevant in the theoretical network of the construct being measured or other external variables that the scores can predict, whereas consequences of testing relates to the robustness of the proposed use of the test scores, including intended benefits, indirect effects, and unintended consequences such as construct underrepresentation or construct irrelevance [14-19]. This study focuses on the evidence collected in the Australian community health setting. Evidence on relations to other variables in this setting has been described by Cheng et al [20]. The aim of this study is to report and evaluate the evidence on test content, response process, and internal structure of the English eHLQ in Australia.

## Methods

### Data Collection

Methods to collect and evaluate the validity evidence were guided by the discussion in the *Standards* and related literature. A mixed methods approach was used with cognitive interviewing conducted to examine evidence on test content and response process, whereas a cross-sectional survey was undertaken for evidence on internal structure. Cognitive interviews were conducted in 2017 at a not-for-profit community health organization in the metropolitan area of Victoria, Australia. The clients of this site, together with clients from 2 private primary care medical clinics in the metropolitan and regional areas, were invited to participate in the cross-sectional survey in 2018.

Eligibility criteria for participation in both activities were clients aged ≥18 years, with or without any health conditions, and able to complete the questionnaire in paper-based format, web-based format, or face-to-face interview. Clients experiencing significant cognitive or mental health issues, who were too clinically unwell, and with insufficient English to complete the questionnaire and who did not have a carer to assist them were excluded.

### Ethics Approval

Ethical approval of the study was obtained from the Deakin University Human Research Ethics Committee (HEAG-H 146_2017).

### Cognitive Interviewing

Cognitive interviewing is commonly used to explore the cognitive process of how people answer survey items [21-23]. It may shed light on how people construct their answers to determine if their thinking matches the item as intended by the test developers, if people experience difficulties when answering the questions, or if the layouts are suitable. The results can also be used to identify response differences across sociocultural groups [17,22,24,25].

Given the qualitative nature of cognitive interviewing, a large sample size is not required but needs to be representative and diverse [14,24]. The process took an iterative approach that involved rounds of testing should issues be identified and the questions needed to be revised [21], with all items tested at least five times or until data saturation [26,27].

Participants were recruited with assistance from the health site, and a plain language information sheet was provided, with written consent requested. Interviews were conducted after participants completed the paper-based format. Participant behavior was observed when they answered the questionnaire. Upon completion, two questions were asked to gain insights into the cognitive process: (1) What were you thinking when you answered this question? (2) Why did you choose this answer? Participants were encouraged to make any further comments about the items or the format. They could be interviewed for all 35 items or part of the questionnaire, mainly for older participants to avoid fatigue and cognitive overload.

Data analysis was conducted using text summary [24]. Content analysis was first reviewed against the item intents of the eHLQ by one of the authors (CC), and further review was undertaken by another author (RHO). Any issues identified for revisions were discussed among all the authors until agreement was reached.

### Cross-sectional Survey for Psychometric Testing

For the cross-sectional survey, clients were recruited at the waiting area and provided with an information sheet. A signed consent form was not requested with the return of the completed questionnaire as implied consent as a strategy to facilitate participation. Apart from self-administration using paper-based or web-based formats, interviews were included to ensure that older people or people with low literacy could participate. Demographic questions including age, sex, postcode, language spoken at home, education, health status, perceived health status, and use of digital services were also collected. Further description of recruitment is described in the study by Cheng et al [20].

Similar to the Danish eHLQ validity testing [10], this study also adopted both the CTT and IRT approaches for psychometric analysis. CTT is the traditional approach based on the assertion that an observed score comprises a true score and an error score [28,29]. This approach usually involves the evaluation of dimensionality, discrimination, and reliability. However, CTT has been criticized for being sample dependent and does not take into account the characteristics of test items and how people at different levels of the construct of interest perform on those items, which is the focus of IRT, the modern approach [28,29]. Hence, both approaches were used in this study to strengthen the collection of evidence.

### Statistical Analysis

#### Overview

Analyses were conducted using three statistical software programs, namely, SPSS (version 25.0; IBM Corp) [30], Mplus (version 8.3; Muthén & Muthén) [31], and IRTPRO (Item Response Theory for Patient-Reported Outcomes; version 4.20; Vector Psychometric Group) [32]. Descriptive statistics were generated for the demographic data, eHLQ scores, and floor and ceiling effects. The presence of floor and ceiling effects may indicate poor discrimination at the minimum or maximum values [33], with effects considered significant if over 15% of participants score in the top (ceiling) or bottom (floor) of a score range [34,35].

#### Missing Values

To deal with missing data for the eHLQ scores, the data set was first examined. If no clear pattern of missingness was found, that is, the missingness could be regarded as completely at random, a 2-step approach would be taken. The first step was to delete cases with more than 50% of missing values to reduce potential bias. The second step was to replace all missing values using the expectation-maximization algorithm imputation in SPSS [30,36,37]. This final data set was used for all psychometric analyses.

#### CTT Analysis

Item difficulty is an item property and is usually conducted as a first step in item analysis in the CTT approach [28,38]. This parameter was calculated as the proportion of survey participants who endorsed disagree and strongly disagree against the proportion of agree and strongly agree [39]. Hence, the higher the proportion responding to disagree and strongly disagree indicated a higher level of difficulty.

To measure reliability, internal consistency and test-retest reliability were evaluated. In addition to the commonly used Cronbach *α* for internal consistency, which has been criticized for producing biased estimates when items do not have equal factor loadings or in case of correlated item errors [39], composite scale reliability calculated through structural equation modeling using Mplus recommended by Raykov [40] was also evaluated. These 2 estimates are expected to be fairly comparable when the set of items is unidimensional, has uncorrelated errors, and has high loadings on the true score [29,40]. The acceptable range of both estimates is from 0.70 to 0.95, with 0.80 generally regarded as good reliability [41]. For test-retest, there is no consensus on the optimal length between time points, and invitations were sent 1 week after the first completion of the questionnaire. Test-retest reliability was evaluated using the intraclass correlation coefficient (ICC) [42]. A minimum of 10 participants is considered adequate for detecting an acceptable ICC of 0.70 with 80% power at a significance level of .05 [43], and a sample size of 30 was estimated.

Following the classical item analysis, CFA was conducted, given that the hypothesized constructs were specified a priori. Similar to the Danish validity testing, the BSEM approach was adopted [10] using Mplus. There is no agreement on the sample size for CFA, which can range from 100 to 400 [33,44], and a sample size of 500 was estimated. Contrary to the traditional frequentist approach using the maximum likelihood estimation procedure, which assumes that measures not related to a latent factor will have zero loadings on that factor, which can easily lead to poor model fit and rejection [29,45], the parameters in BSEM are treated as variables, and this more flexible approach is described as a better reflection of substantive theories [45].

The different parameter specifications in BSEM at the start of an analysis are described as priors, which can be diffuse (noninformative) or informative [45]. Diffuse priors are hypothesized parameters that are fully estimated from the data, whereas informative priors are likely parameter values derived from previous studies, researchers’ theories, or prior beliefs [45]. Informative priors can be applied to cross-loadings and residual covariances. For cross-loading priors, a variance prior of 0.01 means that 95% of the loading variation is within the range of −0.20 to +0.20, which is considered a small loading. Inverse-Wishart priors were used for the covariances among item residuals. The application is to start with a large enough *df* of the inverse-Wishart distribution and gradually lowers the *df* parameter to produce a more flexible model such that the residual covariances are not strictly constrained to zero [46].

A sequence of 1-factor models followed by a 7-factor model (Multimedia Appendix 1) were fitted to the data. Several models with different parameter specifications, using the Danish study as a reference [10], were tested and compared to identify the model of interest, which is a model that is not rejected by the data and is “closest to the CFA model that fits well enough” [46]. To evaluate model fit, Mplus produces a posterior predictive *P* (*PPP*) value and 95% CI for the difference between the observed and replicated chi-square values. A low *PPP* value and positive 95% CI indicate poor fit, whereas a *PPP* value of around .5 and a value of 0 falling close to the middle of the 95% CI indicates excellent fit [45]. Furthermore, a nonsignificant prior-posterior predictive *P* (*PPPP*) value, that is, >.05, indicates that the estimates of the cross-loadings can be considered approximate zero and are thus negligible [47]. Apart from the aforementioned estimates, a model comparison can be conducted by examining the model convergence and information criterion. The model of interest is the model with quicker convergence, that is, when the potential scale reduction is consistently less than 1.05 by the smallest number of iterations, and has a lower discrepancy information criterion [46].

On the basis of the results of the selected 7-factor model, discriminant validity was evaluated using the Fornell-Larcker criteria [48] based on shared variance and average variance extracted (AVE). Shared variance is calculated by squaring the interfactor correlation between 2 factors, and the AVE is generated by the average of the sum of the squared factor loadings of the related items [49]. If the AVE is >0.5, the first Fornell-Larcker criterion that “a factor accounts for more variance in the observed variables associated with it than measurement error or similar external, unmeasured influences” [49] is met. To meet the second criterion about the factor’s association with other factors in the conceptual framework, the AVE of any 2 factors both “have to be greater than the shared variance estimate” between the 2 factors [48,49].

**Figure 1:**
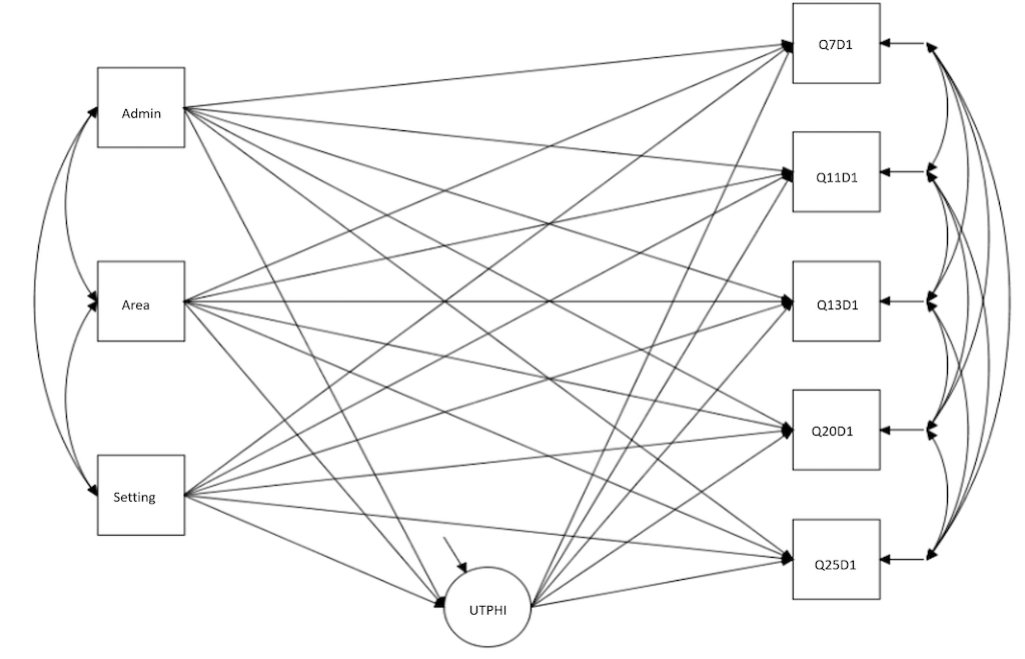
Bayesian multiple-indicators, multiple-causes model for differential item functioning testing with scale 1 of eHealth Literacy Questionnaire as the example. Output from Mplus: Admin: administration format (0=face-to-face interview, 1=paper format); Area: site area, that is, location of participating organization (0=metropolitan, 1=regional); Setting: health setting (0=private clinic, 1=community health); UTPHI: eHealth Literacy Questionnaire scale 1 (using technology to process health information); Q7D1, Q11D1, Q13D1, Q20D1, and Q25D1: eHealth Literacy Questionnaire items.

The BSEM approach is further used to test for differential item functioning (DIF), that is, the stability of measurement across different groups or grouping variables [50,51]. By using the multiple-indicators, multiple-causes (MIMIC) model approach, both DIF and group differences can be detected simultaneously [52]. This method has been used to test for group differences among different demographic groups in the same setting and is described with details in the study by Cheng et al [20]. For this analysis, administration format (paper-based vs face-to-face interviews), site area (metropolitan vs regional area), and health setting (private clinic vs not-for-profit community health) were included as covariates. The web-based format was not included for analysis owing to the limited number of participants (13/525, 2.5%) using this format. See Figure 1 for the Bayesian model for testing DIF with scale 1 as an example. Procedures for model testing and selection are similar to BSEM described earlier, except priors were applied to DIF paths instead of cross-loadings. For the evaluation of DIF, a significant direct effect indicated the presence of DIF, and a 1-tailed *P* value of <.025 was considered significant as no directional hypotheses were set up. If DIF was identified, the estimates were further evaluated based on the model selected. For example, if the model with variance prior of 0.01 was selected and the *PPPP* value was nonsignificant, then estimates within the range of −0.2 to +0.2 could be considered negligible [53]. Group differences were also examined as supplementary results.

#### IRT Analysis

To perform an IRT analysis, 4 assumptions need to be met. The assumptions of unidimensionality (items are measuring the same construct), local independence (each item should not be related except they are measuring the same construct), and item invariance (item parameters are the same across subgroups) can be examined through the CTT methods described in the CTT Analysis section. The assumption of monotonicity (the probability of endorsing an item increases as the trait level increases) is evaluated by examining the test characteristic curves [54]. The sample size requirement for IRT analysis may range from 200 to 500 [54]. For this analysis, the generalized partial credit model, similar to the Danish study, was applied using IRTPRO. Apart from the test characteristic curves, item thresholds, item location, item discrimination, and information functions from the 7 unidimensional IRT models were evaluated. Item response thresholds were evaluated by inspecting the item characteristic curves, where the peak of each response category curve from the lowest (strongly disagree) to the highest (strongly agree) should correspond to the lowest to the highest trait level to demonstrate that an item has an ordered set of response thresholds. For item discrimination, a steeper curve or slope of the item characteristic curves indicates better discrimination between people with different levels of the trait. A higher item discrimination estimate indicates higher discrimination between people with differences in ability [55,56]. For item location estimates, lower estimates represent easier items, as these items are expected to be endorsed by people with lower ability and are expected to be similar to the item difficulty estimates in the CTT analysis [54,57,58]. Finally, an inspection of the information function curve provided information on where an item could precisely measure the underlying trait. This measurement precision is analogous to the reliability in CTT [14]. It also helps to determine if the items of a scale measure the full spectrum of the underlying trait.

## Results

### Cognitive Interviewing

A total of 12 participants were recruited for 2 rounds of cognitive interviews. Of these 12 participants, 8 (67%) were women and 4 (33%) were men, with 58% (7/12) of the participants aged >65 years and 17% (2/12) speaking a language other than English at home. The sample provided a good representation of people from a range of different age groups and cultural backgrounds. The first round with 7 participants identified the term *health technology services* used in the 4 items of scale 7 (*Digital services that suit individual needs*) was confusing. Most participants could not immediately relate the term to digital systems as intended. Following the discussion within the research team, the term was changed to *eHealth systems*. The changed term was tested in a second round with 5 participants, and no further issue was identified. Participants’ understanding of the other items was generally similar to the item intents. For example, for items about using *technology* to find, understand, share, or organize health information, participants could easily link it to the internet, Dr Google, or anything web based. In the item “I use measurements about my body...,” participants thought about how they used results of blood test, body weight, or blood pressure, which was aligned with the item intent.

Despite the diverse backgrounds of participants, no major differences in understanding the items were identified, and all participants found the items easy to read. Recommendations from participants also led to changes in the introductory page to provide examples of technology, health technology, eHealth systems, and health care providers or health professionals. The completion time of the questionnaire ranged from 3 to <7 minutes.

### Psychometric Testing

#### Participant Characteristics

A total of 530 completed questionnaires were collected. On the basis of the treatment of missing values described in the Statistical Analysis section, 5 cases were deleted, leading to a final sample size of 525 for psychometric analyses. The age of participants of the cross-sectional survey ranged from 18 to 94 years, and 61% (320/525) of the participants were women. A total of 33.3% (175/525) of the participants had a university education, and 30.9% (162/535) spoke a language other than English at home. Of the 525 participants, 300 (57.1%) reported having some form of chronic illness. Regarding technology use, of the 525 participants, 151 (28.8%) did not have a computer or laptop, and 131 (25%) did not use email or SMS text messaging (Table 1).

**Table 1:** Characteristics of cross-sectional survey participants (N=525).

Characteristics			Value
Age (years), mean (SD; range)			56.8 (18.6; 18-94)
**Setting, n (%)**			
	Site 1: metropolitan private clinic	204 (38.9)	
	Site 2: metropolitan community health	204 (38.9)	
	Site 3: regional private clinic	117 (22.3)	
**Administration format, n (%)**			
	Paper-based	399 (76)	
	Web-based	13 (2.5)	
	Face-to-face interview	113 (21.5)	
**Sex, n (%)**			
	Female	320 (61)	
	Male	203 (38.7)	
**Education, n (%)**			
	Primary school or below	27 (5.1)	
	Secondary school or below	173 (33)	
	Trade certificate or diploma	141 (26.9)	
	Completed university	175 (33.3)	
**Language at home, n (%)**			
	English	363 (69.1)	
	Other	161 (30.7)	
**Socioeconomic status**^a^, **n (%)**			
	IRSD^b^ 1-4	123 (23.5)	
	IRSD 5-6	111 (21.1)	
	IRSD 7-8	134 (25.5)	
	IRSD 9-10	140 (26.6)	
**Private health insurance, n (%)**			
	Yes	249 (47.4)	
	No	267 (50.9)	
**Longstanding illness (a participant may have >1), n (%)**			
	No	225 (42.9)	
	Arthritis	115 (21.9)	
	Cancer	14 (2.7)	
	Heart disease	90 (17.1)	
	Diabetes	67 (12.8)	
	Respiratory condition	41 (7.8)	
	Anxiety	69 (13.1)	
	Depression	69 (13.1)	
	Other	89 (17)	
**Perceived health status, n (%)**			
	Good to excellent	400 (76.1)	
	Fair to poor	103 (21.5)	
**Ownership of digital device (a participant may have >1), n (%)**			
	Computer or laptop	374 (71.2)	
	Mobile phone or smartphone	459 (87.4)	
	Tablet	241 (45.9)	
Number of devices owned, mean (SD; range)			2.1 (0.9; 0-4)
**Use of digital communication platform (a participant may have >1), n (%)**			
	Email	394 (75)	
	SMS text messaging	398 (75.8)	
	Facebook	266 (50.7)	
	Twitter	30 (5.7)	
	Instagram	104 (19.8)	
	Snapchat	51 (9.7)	
	WhatsApp or WeChat	112 (21.3)	
	Blogging	15 (2.9)	
	Forum/chat room	26 (5)	
Number of platforms used, mean (SD; range)			2.7 (1.8; 0-10)
Looked for web-based information in the last 3 months, n (%)			392 (74.4)
Monitored health digitally, n (%)			183 (34.9)

^a^Socioeconomic status is classified by IRSD10. This index is based on information provided by the Australian Bureau of Statistics [59]. Postcodes are divided into 10 ranks with the higher number indicating more advantaged suburbs.

^b^IRSD: Index of Relative Socio-economic Disadvantage Decile 2016 of Australia.

#### Descriptive Statistics

The mean scale scores ranged from 2.43 (SD 0.57) for scale 7 (*Digital services that suit individual needs*) to 2.95 (SD 0.41) for scale 2 (*Understanding of health concepts and language*; Table 2). Missing values for individual items were <5%, another indication that the items were generally well understood. No floor effect was found, whereas ceiling effects were found for 8 items (range 16.2%-20.8%). These items were related to the use of technology and understanding of health knowledge, suggesting that a substantial proportion of participants were comfortable in using technology and had good knowledge of health (Multimedia Appendix 2).

Observation during cognitive interviewing and the main survey did not identify any issue when people responded to the items for either the paper-based or web-based format. An inspection of the comments marked on the 530 completed questionnaires from the main survey found that 0.03% (15/530) of the participants put a question mark next to some items, indicating that they did not understand those items, while 0.10% (55/530) of the participants provided unclear answers. These results suggested that the items were generally understood, and the 4-point ordinal scale was acceptable.

**Table 2:** eHealth Literacy Questionnaire scale scores (N=525; score range 1-4).

Scale	Value, mean (SD)	Missing data
1. Using technology to process health information	2.59 (0.61)	0
2. Understanding of health concepts and language	2.95 (0.41)	0
3. Ability to actively engage with digital services	2.65 (0.68)	1
4. Feel safe and in control	2.83 (0.49)	5
5. Motivated to engage with digital services	2.63 (0.55)	0
6. Access to digital services that work	2.64 (0.45)	1
7. Digital services that suit individual needs	2.43 (0.57)	11

#### CTT Analysis

A range of item difficulty was found for all scales, reflecting a spectrum of difficulty levels across the relevant constructs. The scale with the smallest range of item difficulty was 7 (*Digital services that suit individual needs*; range 45%-60%). The widest range of item difficulty was observed for scale 4 (*Feel safe and in control*), ranging from 14% to 52%. Scale 7 was also the most *difficult* scale, as the difficulty level of all items was at least 45%, whereas scale 2 (*Understanding of health concepts and language*) was the *easiest* scale with 4 items <20% and the hardest item was 37% (Multimedia Appendix 3).

The chosen 1-factor Bayesian models (with informative priors for residual covariances of *df*=60) had *PPP* values that ranged from 0.19 to 0.24 for the 7 scales with all target loadings statistically significant, establishing evidence of scale homogeneity. Factor loadings were all >0.50, except for item 3 of scale 6 (*Access to digital services that work*) with a loading of 0.45. Residual variances were <0.50, except for 6 items, with item 3 recording the highest estimate of 0.80 (Multimedia Appendix 3).

A subsequent 7-factor model was fitted to the data set with 6 models tested. All models fitted the data well. The model with priors for the variance of cross-loadings set to 0.01 and inverse-Wishart *df* for residual covariances of 150 was chosen as the model of interest (*PPP*=.49, 95% CI for the difference between observed and replicated *χ*^2^ values −101.40 to 108.83, *PPPP*=.92). No statistically significant cross-loadings were found for the chosen model, indicating that all items loaded only on 1 factor (Multimedia Appendix 4). Except for 4 items, all factor loadings of the chosen 7-factor model were >0.50, with item 26 of scale 2 (*Understanding of health concepts and language*) recording the lowest loading of 0.36. In addition, all cross-loadings were less than −0.20 to +0.20; that is, they could be considered approximate zero and negligible (Table 3).

Inspection of the AVE showed that the estimates of 4 scales met the first Fornell-Larcker criterion, whereas 3 scales were <0.50 (scales 2, 4, and 6). Given that these AVE estimates were based on the 7-factor model that allowed for cross-loadings and residual covariances, AVE estimates from the 1-factor models were also calculated, and the AVE estimates for the 7 scales were 0.66, 0.49, 0.72, 0.61, 0.65, 0.47, and 0.74. Hence, the AVE estimates of scales 2 and 6 were still <0.50. The second criterion of the factor’s association with other factors was also not satisfied. On the basis of this criterion, only scale 2 demonstrated good discrimination with scales 4, 6, and 7, and scale 4 had good discrimination with all scales except scale 6. Hence, there might not be sufficient discriminant validity among the scales (Table 4).

For internal consistency, the Cronbach *α* (range .74-.90) and composite scale reliability (range 0.73-0.90) estimates were very similar as expected. All were within the acceptable range, whereas scales 1, 3, 4, 5, and 7 had estimates >0.80, indicating good internal consistency. For test-retest, 42 participants completed the retest and ICC ranged from 0.72 to 0.95, suggesting good test-retest reliability (Multimedia Appendix 3).

The Bayesian MIMIC models for testing DIF for administration format, site area, and health setting achieved a good model fit. The model with a DIF path of 0.01 was chosen as the model of interest for the 7 scales with *PPP* ranging from .19 (scale 6) to .35 (scale 7), and all *PPPP*s were nonsignificant. No statistically significant effects of site area and health setting on the items were found. The administration format was found to have a statistically direct effect on 5 items, indicating possible DIF. However, all estimates were within the acceptable range of −0.2 to +0.2 and therefore considered negligible (Multimedia Appendix 5).

For group differences, no significant differences were found for site area and health setting, but group differences were identified for the administration format with the self-administered paper-based format scoring higher than face-to-face interviews for scales 1 (*Using technology to process health information*), 3 (*Ability to actively engage with digital services*), 5 (*Motivated to engage with digital services*), and 7 (*Digital services that suit individual needs*; Table 5). Further analysis using nonparametric tests was undertaken to explore if the 2 groups had significant differences in terms of age, education, and technology use, with a significance level set at <.05. A Mann-Whitney *U* test indicated significant difference in age for interview (median 75; n=109) and paper format (median 51; n=387; *U*=7881.50; *z*=−10.00; *P*<.001), with participants interviewed being older than those completing the self-administered paper format. A chi-square test for independence indicated a significant association between education and administration format, *χ*^2^_1_=0.4 (n=503), *P*<.001. Among the participants being interviewed, 36.7% (40/109) did not complete secondary school, whereas only 13.2% (51/387) of participants completing the paper format did not complete secondary school. A significant difference in the number of devices was also found for interview (median 2; n=111) and paper format (median 2; n=390; *U*=20,328.50; *z*=6.86; *P*<.001), with more devices for participants using the self-administered paper format than for participants being interviewed.

**Table 3:** Factor loadings of the eHealth Literacy Questionnaire 7-factor Bayesian confirmatory factor analysis model with priors for cross-loadings of 0.01 and residual covariances of 150^a^.

Item^b^		1. Using technology	2. Health concepts	3. Ability	4. Feel safe	5. Motivated	6. Access	7. Suit needs
**1. Using technology to process health information**								
	Q7D1	*0.94* ^c^	0.02	0.02	0.00	−0.08	−0.06	−0.06
	Q11D1	*0.89*	0.03	−0.01	−0.01	−0.02	−0.04	−0.08
	Q13D1	*0.59*	−0.02	−0.03	0.02	0.08	0.06	0.05
	Q20D1	*0.49*	−0.03	−0.01	0.02	0.05	0.06	0.08
	Q25D1	*0.61*	−0.01	0.02	0.01	0.03	0.04	0.06
**2. Understanding of health concepts and language**								
	Q5D2	0.06	*0.52*	0.03	0.00	0.04	0.01	0.01
	Q12D2	0.02	*0.70*	0.01	0.02	−0.02	−0.03	−0.03
	Q15D2	−0.04	*0.51*	−0.02	0.03	−0.01	0.03	0.02
	Q21D2	−0.03	*0.67*	−0.01	−0.01	−0.03	−0.02	−0.02
	Q26D2	0.02	*0.36*	−0.00	−0.02	0.05	0.04	0.04
**3. Ability to actively engage with digital service**								
	Q4D3	0.00	−0.00	*0.68*	0.04	0.03	0.03	0.03
	Q6D3	0.02	0.01	*0.88*	0.03	−0.02	−0.04	−0.05
	Q8D3	0.03	0.02	*0.62*	0.01	0.03	0.02	0.03
	Q17D3	0.00	−0.01	*0.88*	−0.02	−0.03	−0.03	−0.04
	Q32D3	−0.03	0.01	*0.74*	−0.04	0.01	0.03	0.07
**4. Feel safe and in control**								
	Q1D4	0.02	0.00	0.01	*0.67*	−0.01	−0.02	−0.03
	Q10D4	0.05	0.02	0.01	*0.67*	0.04	0.01	0.00
	Q14D4	0.04	0.05	0.02	*0.40*	0.05	0.05	0.03
	Q22D4	−0.03	−0.02	−0.01	*0.86*	−0.03	−0.01	0.00
	Q30D4	−0.01	−0.01	0.01	*0.74*	0.01	0.02	0.04
**5. Motivated to engage with digital services**								
	Q2D5	−0.04	−0.02	−0.01	0.02	*0.76*	−0.01	−0.02
	Q19D5	0.04	0.02	0.02	−0.04	*0.71*	−0.00	0.00
	Q24D5	−0.02	0.01	−0.02	0.05	*0.67*	0.02	0.01
	Q27D5	0.00	0.01	−0.01	0.01	*0.74*	−0.01	−0.02
	Q35D5	0.04	0.00	0.03	−0.01	*0.72*	−0.00	0.00
**6. Access to digital services that work**								
	Q3D6	-0.11	0.02	−0.05	0.06	−0.08	*0.59*	−0.08
	Q9D6	0.13	−0.00	0.08	−0.03	0.05	*0.40*	0.05
	Q16D6	−0.11	0.02	−0.04	0.05	−0.05	*0.65*	−0.02
	Q23D6	0.05	−0.03	0.01	0.00	0.03	*0.61*	0.01
	Q29D6	0.00	−0.01	−0.01	−0.01	0.02	*0.61*	−0.00
	Q34D6	0.12	0.01	0.08	−0.07	0.07	*0.48*	0.07
**7. Digital services that suit individual needs**								
	Q18D7	0.05	0.02	0.04	−0.02	−0.02	−0.03	*0.74*
	Q28D7	0.00	−0.03	−0.02	0.01	0.03	0.01	*0.78*
	Q31D7	−0.09	0.01	−0.05	0.07	−0.04	0.03	*0.85*
	Q33D7	0.02	0.00	0.04	−0.04	−0.00	−0.02	*0.85*

^a^Model fit: posterior predictive *P*=0.49, 95% CI for the difference between observed and replicated χ^2^ values −101.40 to 108.83, prior-posterior predictive *P*=.92.

^b^See Multimedia Appendix 2 for truncated items.

^c^Italicized values indicate statistically significant factor loadings (*P*<.05) with standardized estimates reported.

**Table 4:** Interfactor correlations (below diagonal), average variance extracted (diagonal), and shared variance estimates (above diagonal) for the 7 eHealth Literacy Questionnaire scales.

Scale	1. Use tech	2. Health concepts	3. Ability	4. Feel safe	5. Motivated	6. Access	7. Suit needs
1. Using technology to process health information	0.53^a^	0.37^b^	0.90^b^	0.06	0.84^b^	0.38^b^	0.56^b^
2. Understanding of health concepts and language	0.61	0.32^a^	0.38^b^	0.22	0.34^b^	0.25	0.21
3. Ability to actively engage with digital services	0.95	0.62	0.59^a^	0.04	0.72^b^	0.34 ^b^	0.61^b^
4. Feel safe and in control	0.25	0.47	0.21^c^	0.47^a^	0.12	0.34^b^	0.19
5. Motivated to engage with digital services	0.91	0.58	0.85	0.35	0.52^a^	0.63^b^	0.69^b^
6. Access to digital services that work	0.62	0.50	0.58	0.58	0.80	0.32^a^	0.75^b^
7. Digital services that suit individual needs	0.75	0.46	0.78	0.43	0.83	0.87	0.65^a^

^a^These values indicated average variance extracted by each latent variable.

^b^These values indicate that latent variable shared variance estimates exceed the average variance extracted of either or both variables.

^c^Statistically not significant interfactor correlation (*P*>.05).

**Table 5:** Estimated effects of administration format, site area, and health setting on the 7 eHealth literacy latent variables.

Scale	Admin format^a,b^	Site area^a,c^	Health setting^a,d^
1. Using technology to process health information	*0.38 (0.05)* ^e^	0.02 (0.06)	0.10 (0.06)
2. Understanding of health concepts and language	−0.02 (0.07)	−0.00 (0.07)	0.05 (0.08)
3. Ability to actively engage with digital services	*0.39 (0.05)*	−0.02 (0.05)	0.07 (0.06)
4. Feel safe and in control	−0.03 (0.06)	0.12 (0.06)	−0.04 (0.07)
5. Motivated to engage with digital services	*0.25 (0.05)*	0.03 (0.06)	0.10 (0.06)
6. Access to digital services that work	0.02 (0.06)	0.02 (0.06)	−0.02 (0.07)
7. Digital services that suit individual needs	*0.22 (0.06)*	−0.01 (0.06)	0.06 (0.07)

^a^Standardized estimates reported; posterior SD for estimates shown in parentheses.

^b^Administration format code: 0=interview, 1=paper.

^c^Site area code: 0=metropolitan, 1=regional.

^d^Health setting code: 0=private clinic, 1=community health.

^e^Italicized values indicate statistically significant differences, significant if *P*<.025 (1-tailed).

#### IRT Analysis

The results of the 1-factor Bayesian models with all significant targeted factor loadings provided evidence of unidimensionality and local independence. Item invariance was supported by the testing of DIF for administration format, site area, and health setting. Measurement invariance across subgroups, including age, sex, education, language spoken at home, and information and communication technology use, was also established and reported by Cheng et al [20]. Hence, the results of the CTT analysis confirmed 3 of the 4 assumptions for IRT analysis. For the final assumption of monotonicity, the test characteristic curves were examined, confirming that the probability of endorsing an item increased as the trait level increased.

Visual inspection of the item characteristic curves showed distinct peaks for the response categories along the continuum of the latent trait for the most likely responses, indicating ordered thresholds for all items (Multimedia Appendix 6). The item discrimination parameters demonstrated that items within each scale had different sensitivities to different levels of ability. All slopes of the item characteristic curves were steep, with the steepest slope observed for item 33 of scale 7 (*Digital services that suit individual needs*), which also had the highest item discrimination parameter of 5.56. The items with lower discrimination parameters among all items were item 3 of scale 6 (*Access to digital services that work*; 0.86) and item 26 of scale 2 (*Understanding health concepts and language*; 0.88). However, the item characteristic curves of both items were still considered steep. The item location parameters also showed that items had different levels of difficulty within each scale but were not evenly distributed for scales 2 and 4. Items 15 and 21 of scale 2 (*Understanding of health concepts and language*) had very similar item location parameters of −1.19 and −1.18, respectively. Scale 4 (*Feel safe and in control*) also had item location parameters of −0.58 for item 10 and −0.54 for item 22. The results were generally in line with the item difficulty indexes from the CTT analysis (Multimedia Appendix 3). Furthermore, the information function curves were evaluated for reliability. All items provided precise information at different levels of the latent trait, except item 14 of scale 4 (*Feel safe and in control*), which provided very low information across all levels of the trait (Multimedia Appendix 6).

## Discussion

### Principal Findings

This study collected and examined validity evidence based on test content, response process, and internal structure of the eHLQ in the Australian community health setting. Items and formats were easy to read and use, and items were understood as intended. The Bayesian CFA and IRT analyses confirmed the robustness of the internal structure. However, discriminant validity based on estimates of the 7-factor BSEM was not well established and will require further investigation.

The cognitive interviews were successful in identifying 1 confusing term, which was revised, and the introductory page of the questionnaire was also improved. The results combined with observation during interviews and the survey as well as the limited number of missing values provided a wealth of information in support of the validity evidence on test content and response process of the eHLQ.

The final sample size of 525 of the cross-sectional survey provided an adequate sample size for both the CTT and IRT analyses. Although the sample had more women and university-educated participants than the Australian national averages, the sociodemographic characteristics of the participants still reflected a generally diverse sample. Nevertheless, a quarter of the sample did not use email or look for web-based information, showing that people with limited use of technology or eHealth were represented in the survey. This would ensure that the validity testing results of the eHLQ were also applicable to people with potentially lower eHealth literacy. In addition, the group differences in the 4 scales identified in the administration format for paper-based and interviews further pointed to the results that these 2 groups were significantly different in terms of age, education, and technology use. As such, the purpose of an interview option as a recruitment strategy to include older people or people with lower literacy was fulfilled. By contrast, evidence of measurement invariance across the 2 formats confirmed that responses were not influenced by interviewing bias or social desirability. Given a separate analysis of this sample found that older people also scored lower on the same 4 scales [20], the findings of the group differences between the 2 administration formats are not surprising. However, future studies may consider examining if such group differences continue to persist when the interview option is provided at random. The identified group differences also imply that the interview option should always be available such that older people or people with lower literacy are included in future eHealth literacy research to ensure that they are not being left behind in the age of digital health.

A rigorous assessment of the internal structure was undertaken using both the CTT and IRT approaches to ensure that different aspects of validity and reliability of the eHLQ data were investigated. For the CTT analysis, the Bayesian approach of applying informative priors was used. Although this modern approach may involve more steps in testing model fit, it allows for the hypothesis of approximate zeros for model parameters. Instead of being constrained to exact zeros, as in the traditional structural equation modeling approach, the approach provides a better approximation of the real world. As such, the seven 1-factor models were found to fit the data well, confirming scale homogeneity, while factor loadings and residual variances were acceptable. Estimates of internal consistency reliability were good for all scales, although scale 2 (*Understanding of health concepts and language*) and scale 6 (*Access to digital services that work*) were low but still fell within the acceptable range. Test-retest reliability was also good for all scales, indicating that the eHLQ produces stable and consistent results.

The characteristics of the test and items in accurately measuring eHealth literacy were further supported by the IRT analysis. The test and item characteristic curves demonstrated that participants with higher eHealth literacy were more likely to endorse items with agree and strongly agree. The information function curves indicated that the items could gather reliable and precise information across different levels of the underlying trait. Estimates further showed that the items had generally high sensitivity in discriminating participants with different levels of eHealth literacy. The item locations also supported the fact that the items represented different levels of difficulty. This is further verified with the item difficulty indexes from the CTT analysis, which showed the 2 estimates displaying very similar pattern, further strengthening the evidence that the items generally represented a range of difficulty levels of the latent factor. The use of MIMIC models also found no or negligible DIF for administration format, site area, and health setting, confirming measurement equivalence of the items across formats and settings.

Although it is noted that the Australian results are generally similar to the Danish validity testing results reported by Kayser et al [10], a comparison of the item location results found otherwise. The 2 results are in contradiction such that the easiest item in the Australian main sample is the hardest item in the Danish context for most of the scales. For example, in scale 1 (*Using technology to process health information*), item 7 is the easiest item for the Australian sample, but it is the hardest item for the Danish data set. As the Danish study does not report on CTT item difficulty estimates, it cannot be used to calibrate with the Australian results. This may be due to people’s different practices in using and accessing digital health between the 2 countries, as Denmark has much more regular users of web-based health systems than Australia. The real reason behind the differences is difficult to speculate and future investigation (eg, using cognitive interviewing specifically focused on the levels of response to items with contrasting difficulties in the 2 countries) may shed more light on the discrepancy.

Following the 1-factor models, a subsequent 7-factor model using informative priors for cross-loadings and residual covariances demonstrated excellent model fit of the factor structure, as hypothesized by the questionnaire developers. All target loadings were significant with acceptable factor loadings, and there was also no significant cross-loading for the chosen model of interest. Although the chosen model of interest has informative priors different from the chosen model of the Danish validity testing, the Australian data analyses generally replicate the Danish results, strengthening the evidence of the internal structure of the eHLQ.

A possible weakness in the psychometric properties of the eHLQ may be its discriminant validity. The AVE estimates suggested a lack of clear discrimination among all the scales except for scale 2 (*Understanding of health concepts and language*) and scale 4 (*Feel safe and in control*). Although AVE estimates were not investigated in the Danish validity testing, the high interfactor correlations between scales 1 and 5 and scales 6 and 7 in the Danish validity testing also suggested possible insufficient discrimination among those factors, and it was speculated that there might be some causal relationships among these scales [10]. However, the test developers argued that content analysis of the views of patients and professionals during development confirmed that these factors were indeed different constructs and decided to keep the 7 dimensions in the final model. Further investigation of discriminant validity is warranted in future validity testing of the tool.

This study provided robust validity evidence of inferences drawn from the eHLQ when used in the diverse Australian community health settings. As this study was undertaken before the COVID-19 pandemic, which sees an increased acceptance and use of telehealth [60] as well as the widespread of misinformation and disinformation on social media [1], the eHLQ will be a useful tool for health care providers, researchers, digital health developers, and policy makers to better understand the eHealth literacy needs of individual patients and different population groups. The insights gained will help develop, implement, and evaluate digital health interventions that suit the needs of users to promote health and equity.

### Limitations

A possible limitation to the validity evidence is that the sample involved only participants who spoke and understood English well. Although the eHLQ is one of the first questionnaires developed simultaneously in 2 languages to minimize cultural references, both languages are from Western culture with generally well-developed national health care systems. How the psychometric properties perform in other cultural groups and countries is not clear. Future research on the eHLQ should include validity testing in cross-cultural settings including in different contexts and use. The Danish validity testing study was undertaken in the community setting involving the general population. However, this study only included people attending community health services. Future testing of the eHLQ in other Australian settings may strengthen the validity evidence of the tool for the general population.

### Conclusions

The evidence presented in this study suggests that the eHLQ is a tool with robust psychometric properties. There is support for test content, and the items are understood as intended. Although there are potential weaknesses in discriminant validity, it is reasonable to suggest that the items can provide valid and reliable assessment of the 7 constructs of eHealth literacy in the diverse Australian health settings. The eHLQ is ready to be used to identify eHealth literacy strengths and challenges and assist the development of digital health interventions to ensure that people with limited digital access and skills are not being left behind.

## Appendix

Multimedia Appendix 1 Bayesian structural equation model of the eHealth Literacy Questionnaire with no prior. Output from Mplus.

Multimedia Appendix 2 Descriptive statistics of the eHealth Literacy Questionnaire items.

Multimedia Appendix 3 Psychometric properties of the eHealth Literacy Questionnaire single scales.

Multimedia Appendix 4 Bayesian model fit information of the eHealth Literacy Questionnaire 7-factor models.

Multimedia Appendix 5 Estimates for the direct effect of eHealth Literacy Questionnaire items on administration format, site area, and health setting.

Multimedia Appendix 6 Item characteristic curves and information function curves of the eHealth Literacy Questionnaire items (Item Response Theory for Patient-Reported Outcomes outputs).

## Abbreviations

AVE: average variance extracted
BSEM: Bayesian structural equation modeling
CFA: confirmatory factor analysis
CTT: classical test theory
DIF: differential item functioning
eHLQ: eHealth Literacy Questionnaire
ICC: intraclass correlation coefficient
IRT: item response theory
MIMIC: multiple-indicators, multiple-causes
PPP: posterior predictive P
PPPP: prior-posterior predictive P
