# Supplementary material for: Validity Evidence of the eHealth Literacy Questionnaire (eHLQ) Part 2: Mixed Methods Approach to Evaluate Test Content, Response Process, and Internal Structure in the Australian Community Health Setting
Source: J Med Internet Res. 2022 Mar 8;24(3):e32777. doi: 10.2196/32777 (PMC8941428; doi:10.2196/32777)
Supplement: Multimedia Appendix 1 [file jmir_v24i3e32777_app1.docx]

**Multimedia Appendix 1:** Bayesian structural equation model of the eHealth Literacy Questionnaire with no prior. Output from Mplus.


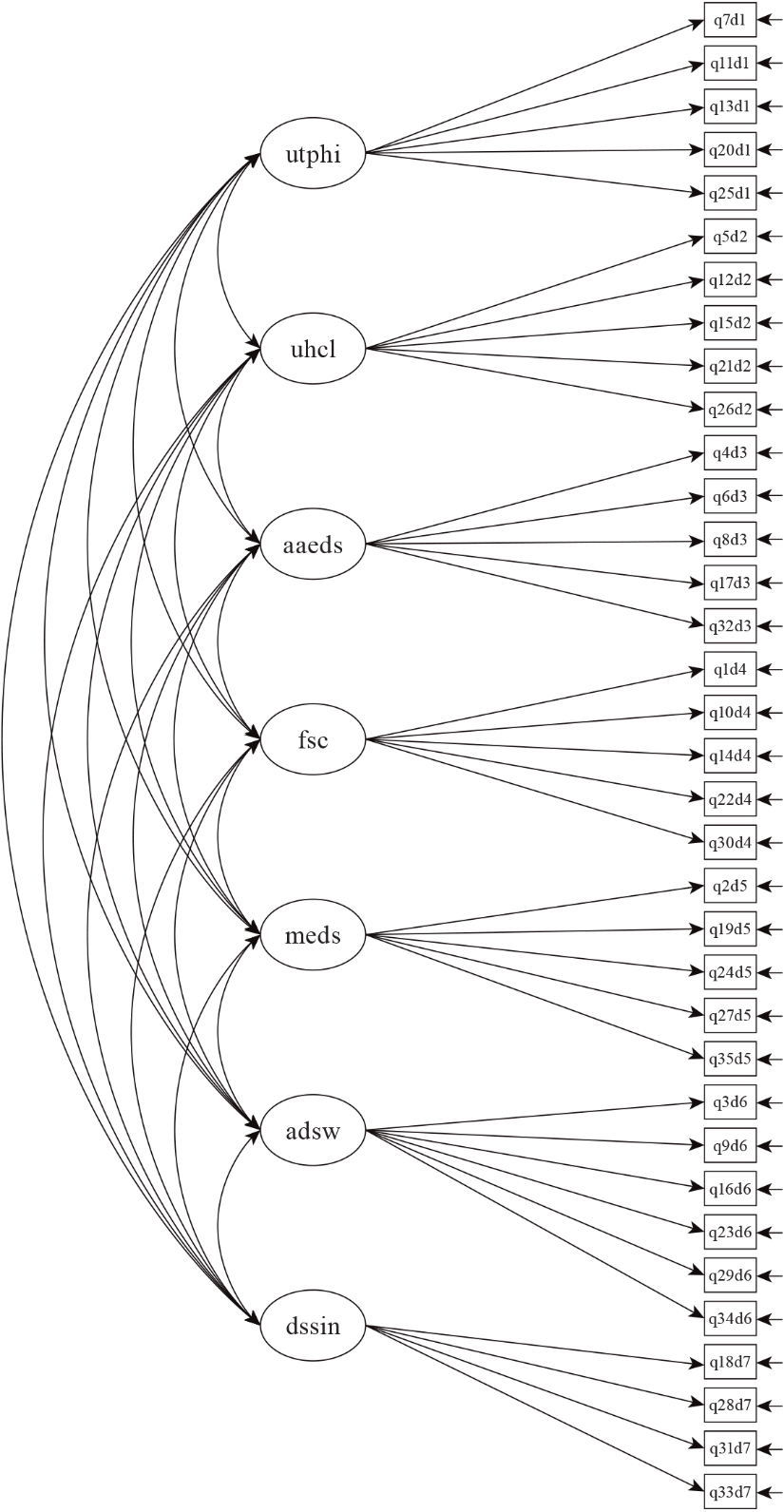


circle = latent variables; square = observed variables (questionnaire items).

utphi = using technology to process health information; uhcl = understanding of health concepts and language; aaeds = ability to actively engage with digital services; fsc = feel safe and in control; meds = motivated to engage with digital services; adsw = access to digital services that work; dssin = digital services that suit individual needs.
