# Supplementary material for: Validity Evidence of the eHealth Literacy Questionnaire (eHLQ) Part 2: Mixed Methods Approach to Evaluate Test Content, Response Process, and Internal Structure in the Australian Community Health Setting
Source: J Med Internet Res. 2022 Mar 8;24(3):e32777. doi: 10.2196/32777 (PMC8941428; doi:10.2196/32777)
Supplement: Multimedia Appendix 2 [file jmir_v24i3e32777_app2.docx]

**Multimedia Appendix 2:** Descriptive statistics of the eHealth Literacy Questionnaire items.

| **Scale/Item** | | **Missing (%)** | **Floor effect (%)** | **Ceiling effect (%)** | **Mean (SD)** |
| --- | --- | --- | --- | --- | --- |
| **1. Using technology to process health information** | | | | | |
| Q7 | I use technology to find… | 0.8 | 6.1 | **20.8** | 2.9 (0.8) |
| Q11 | I often use technology… | 1.0 | 6.9 | **17.1** | 2.8 (0.8) |
| Q13 | Technology helps me… | 1.9 | 6.5 | 9.0 | 2.6 (0.8) |
| Q20 | I use technology to share… | 1.0 | 8.4 | 4.6 | 2.3 (0.7) |
| Q25 | I use technology to organize… | 1.1 | 8.4 | 4.6 | 2.4 (0.7) |
| **2. Understanding of health concepts and language** | | | | | |
| Q5 | The knowledge I have helps me… | 0.6 | 1.1 | **16.2** | 3.0 (0.6) |
| Q12 | I have enough information… | 1.1 | 0.8 | 14.9 | 3.0 (0.6) |
| Q15 | I understand medical results… | 2.3 | 0.4 | 12.2 | 3.0 (0.5) |
| Q21 | Overall, I understand how… | 1.5 | 0.4 | **17.5** | 3.1 (0.6) |
| Q26 | I use measurements about… | 0.8 | 3.0 | 9.1 | 2.7 (0.7) |
| **3. Ability to actively engage with digital services** | | | | | |
| Q4 | I know how to use technology… | 1.0 | 7.0 | **16.4** | 2.7 (0.8) |
| Q6 | I know how to make… | 1.1 | 5.9 | **18.1** | 2.8 (0.8) |
| Q8 | I can enter data into… | 2.5 | 10.1 | 10.7 | 2.5 (0.8) |
| Q17 | I quickly learn how to find… | 0.6 | 8.0 | **16.8** | 2.7 (0.9) |
| Q32 | I easily learn to use new… | 1.7 | 7.4 | 9.7 | 2.6 (0.8) |
| **4. Feel safe and in control** | | | | | |
| Q1 | I am sure that my health data… | 1.5 | 2.1 | **20.0** | 3.0 (0.6) |
| Q10 | My electronic healthcare data… | 4.8 | 1.9 | 9.3 | 2.9 (0.6) |
| Q14 | I have a clear understanding… | 1.9 | 5.1 | 5.0 | 2.5 (0.7) |
| Q22 | I am sure that only authorized… | 3.2 | 2.7 | 13.3 | 2.9 (0.7) |
| Q30 | I am confident that healthcare… | 3.4 | 1.5 | 10.9 | 2.9 (0.6) |
| **5. Motivated to engage with digital services** | | | | | |
| Q2 | Technology makes me… | 1.1 | 4.6 | 9.9 | 2.7 (0.7) |
| Q19 | I find technology helps me… | 1.5 | 4.2 | 7.8 | 2.7 (0.7) |
| Q24 | I find I get better services… | 3.0 | 4.2 | 4.8 | 2.4 (0.7) |
| Q27 | Technology improves… | 1.0 | 5.0 | 8.0 | 2.6 (0.7) |
| Q35 | I find technology useful… | 1.1 | 4.0 | 9.0 | 2.7 (0.7) |
| **6. Access to digital services that work** | | | | | |
| Q3 | Information about my health… | 1.1 | 2.1 | 12.8 | 2.9 (0.6) |
| Q9 | My healthcare providers… | 2.9 | 3.8 | 6.9 | 2.6 (0.7) |
| Q16 | My health data are available… | 3.0 | 3.8 | 5.0 | 2.5 (0.7) |
| Q23 | All the health technology I use… | 4.4 | 7.0 | 3.6 | 2.4 (0.7) |
| Q29 | Most of my healthcare… | 2.3 | 1.9 | 7.2 | 2.8 (0.6) |
| Q34 | I have access to health… | 3.0 | 3.6 | 5.7 | 2.6 (0.7) |
| **7. Digital services that suit individual needs** | | | | | |
| Q18 | I find that eHealth systems… | 4.0 | 9.1 | 2.9 | 2.4 (0.7) |
| Q28 | I find eHealth systems seem to… | 4.6 | 7.2 | 2.1 | 2.4 (0.7) |
| Q31 | I find eHealth systems are… | 4.4 | 5.5 | 3.8 | 2.5 (0.7) |
| Q33 | eHealth systems provide me… | 3.4 | 6.1 | 3.8 | 2.5 (0.7) |

Items are truncated. Please contact the authors for full items.

Bold=over 15%.
