# Supplementary material for: Validity Evidence of the eHealth Literacy Questionnaire (eHLQ) Part 2: Mixed Methods Approach to Evaluate Test Content, Response Process, and Internal Structure in the Australian Community Health Setting
Source: J Med Internet Res. 2022 Mar 8;24(3):e32777. doi: 10.2196/32777 (PMC8941428; doi:10.2196/32777)
Supplement: Multimedia Appendix 3 [file jmir_v24i3e32777_app3.docx]

**Multimedia Appendix 3:** Psychometric properties of the eHealth Literacy Questionnaire single scales.

|  | **CTT analyses** |  |  | **IRT analyses** |  |
| --- | --- | --- | --- | --- | --- |
| **Items** | **Factor loading^a^**  **(95% CI^b^)** | **Residual variance^a^**  **(95% CI^b^)** | **Item difficulty (95% CI)** | **Item location (95% CI)** | **Item discrimination (95% CI)** |
| **1. Using technology to process health information** | | | | | |
| Q7^c^ | 0.87  (0.80 – 0.92) | 0.25  (0.16 – 0.36) | 0.29  (0.25 – 0.33) | -0.48  (-0.60 – -0.36) | 3.66  (2.78 – 4.54) |
| Q11 | 0.88  (0.81 – 0.92) | 0.24  (0.15 – 0.34) | 0.34  (0.30 – 0.38) | -0.36  (-0.48 – -0.24) | 3.90  (2.90 – 4.90) |
| Q13 | 0.80  (0.73 – 0.86) | 0.36  (0.26 – 0.47) | 0.44  (0.40 – 0.48) | -0.15  (-0.31 – 0.01) | 2.00  (1.59 – 2.41) |
| Q20 | 0.71  (0.60 – 0.79) | 0.50  (0.37 – 0.64) | 0.63  (0.59 – 0.67) | 0.28  (0.10 – 0.46) | 1.43  (1.12 – 1.74) |
| Q25 | 0.78  0.69 – 0.84) | 0.40  (0.29 – 0.52) | 0.57  (0.53 – 0.62) | 0.21  (0.05 – 0.37) | 1.87  (1.46 – 2.28) |
| *Model fit: PPP = .21; 95% CI for X^2^ difference = -11.99 – 31.45*  *Cronbach’s Alpha = .86*  *Composite Scale Reliability (95% CI) = 0.87 (0.85 – 0.88)*  *Test-retest Intraclass Correlation Coefficient (ICC) (95% CI) = 0.95 (0.90 – 0.97)* | | | | | |
| **2. Understanding of health concepts and language** | | | | | |
| Q5 | 0.67  (0.50 – 0.80) | 0.55  (0.37 – 0.75) | 0.17  (0.14 – 0.20) | -1.04  (-1.29 – -0.79) | 1.42  (1.07 – 1.77) |
| Q12 | 0.73  (0.61 – 0.83) | 0.46  (0.31 – 0.63) | 0.14  (0.12 – 0.18) | -0.94  (-1.16 – -0.72) | 2.50  (1.79 – 3.21) |
| Q15 | 0.64  (0.50 – 0.75) | 0.59  (0.43 – 0.75) | 0.15  (0.12 – 0.18) | -1.19  (-1.56 – -0.82) | 1.58  (1.19 – 1.97) |
| Q21 | 0.73  (0.61 – 0.82) | 0.47  (0.33 – 0.63) | 0.12  (0.09 – 0.15) | -1.18  (-1.47 – -0.89) | 2.26  (1.57 – 2.95) |
| Q26 | 0.71  (0.54 – 0.84) | 0.49  (0.29 – 0.71) | 0.37  (0.33 – 0.41) | -0.53  (-0.78 – -0.28) | 0.88  (0.64 – 1.12) |
| *Model fit: PPP = .21; 95% CI for X^2^ difference =* -*12.30 – 31.36*  *Cronbach’s Alpha = .74*  *Composite Scale Reliability (95% CI) = 0.73 (0.70 – 0.77)*  *Test-retest ICC (95% CI) = 0.72 (0.48 – 0.85)* | | | | | |
| **3. Ability to actively engage with digital services** | | | | | |
| Q4 | 0.82  (075 – 0.88) | 0.33  (0.23 – 0.44) | 0.37  (0.33 – 0.41) | -0.34  (-0.56 – -0.12) | 2.13  (1.72 – 2.54) |
| Q6 | 0.89  (0.85 – 0.93) | 0.21  (0.15 – 0.28) | 0.34  (0.30 – 0.38) | -0.40  (-0.60 – -0.20) | 4.56  (3.07 – 6.05) |
| Q8 | 0.80  (0.73 – 0.86) | 0.36  (0.25 – 0.47) | 0.52  (0.48 – 0.56) | 0.00  (-0.20 – 0.19) | 1.85  (1.50 – 2.20) |
| Q17 | 0.87  (0.81 – 0.92) | 0.24  (0.16 – 0.34) | 0.41  (0.37 – 0.45) | -0.25  (-0.43 – -0.07) | 3.25  (2.29 – 4.21) |
| Q32 | 0.84  (0.78 – 0.89) | 0.30  (0.21 – 0.40) | 0.43  (0.39 – 0.47) | -0.14  (-0.34 – 0.06) | 2.82  (2.06 – 3.58) |
| *Model fit: PPP = .21; 95% CI for X^2^ difference = -12.28 – 31.44*  *Cronbach’s Alpha = .90*  *Composite Scale Reliability (95% CI) = 0.90 (0.88 – 0.91)*  *Test-retest ICC (95% CI) = 0.90 (0.81 – 0.95)* | | | | | |
|  | **CTT analyses** |  |  | **IRT analyses** |  |
| **Items** | **Factor loading^a^**  **(95% CI^b^)** | **Residual variance^a^**  **(95% CI^b^)** | **Item difficulty (95% CI)** | **Item location (95% CI)** | **Item discrimination (95% CI)** |
| **4. Feel safe and in control** | | | | | |
| Q1 | 0.77  (0.67 – 0.85) | 0.41  (0.29 – 0.56) | 0.14  (0.12 – 0.18) | -0.90  (-1.10 – -0.70) | 2.15  (1.72 – 2.58) |
| Q10 | 0.81  (0.73 – 0.86) | 0.35  (0.26 – 0.47) | 0.23  (0.20 – 0.27) | -0.58  (-0.76 – -0.40) | 2.44  (1.93 – 2.95) |
| Q14 | 0.66  (0.52 – 0.78) | 0.56  (0.40 – 0.73) | 0.52  (0.47 – 0.56) | 0.03  (-0.21 – 0.27) | 1.05  (0.81 – 1.29) |
| Q22 | 0.87  (0.80 – 0.91) | 0.25  (0.17 – 0.36) | 0.24  (0.21 – 0.28) | -0.54  (-0.70 – -0.38) | 4.43  (3.37 – 5.49) |
| Q30 | 0.80  (0.72 – 0.86) | 0.37  (0.27 – 0.49) | 0.18  (0.15 – 0.21) | -0.68  (-0.84 – -0.52) | 3.13  (2.17 – 4.09) |
| *Model fit: PPP = .22; 95% CI for X^2^ difference = -12.57 – 30.64*  *Cronbach’s Alpha = .83*  *Composite Scale Reliability (95% CI) = 0.84 (0.81 – 0.86)*  *Test-retest ICC (95% CI) = 0.84 (0.60 – 0.91)* | | | | | |
| **5. Motivated to engage with digital services** | | | | | |
| Q2 | 0.75  (0.66 – 0.83) | 0.43  (0.31 – 0.56) | 0.32  (0.28 – 0.36) | -0.37  (-0.64 – -0.10) | 1.68  (1.35 – 2.01) |
| Q19 | 0.81  (0.74 – 0.87) | 0.34  (0.24 – 0.46) | 0.36  (0.32 – 0.40) | -0.27  (-0.52 – -0.02) | 2.49  (2.96 – 3.02) |
| Q24 | 0.79  (0.71 – 0.85) | 0.38  (0.28 – 0.49) | 0.59  (0.55 – 0.63) | 0.05  (-0.20 – 0.30) | 2.67  (1.94 – 3.40) |
| Q27 | 0.83  (0.77 – 0.89) | 0.30  (0.21 – 0.42) | 0.39  (0.35 – 0.43) | -0.21  (-0.45 – 0.03) | 2.79  (1.99 – 3.59) |
| Q35 | 0.83  (0.77 – 0.89) | 0.31  (0.22 – 0.41) | 0.37  (0.33 – 0.41) | -0.30  (-0.54 – -0.06) | 2.78  (1.84 – 3.72) |
| *Model fit: PPP = .21; 95% CI for X^2^ difference = -12.13 – 31.22*  *Cronbach’s Alpha = .86*  *Composite Scale Reliability (95% CI) = 0.86 (0.84 – 0.88)*  *Test-retest ICC (95% CI) =0.95 (0.90 – 0.97)* | | | | | |
| **6. Access to digital services that work** | | | | | |
| Q3 | 0.45  (0.20 – 0.64) | 0.80  (0.60 – 0.96) | 0.23  (0.20 – 0.27) | -0.95  (-1.26 – -0.64) | 0.86  (0.62 – 1.10) |
| Q9 | 0.73  (0.60 – 0.82) | 0.47  (0.32 – 0.64) | 0.44  (0.40 – 0.49) | -0.21  (-0.39 – -0.03) | 1.39  (1.08 – 1.70) |
| Q16 | 0.68  (0.52 – 0.80) | 0.53  (0.36 – 0.73) | 0.50  (0.46 – 0.55) | -0.05  (-0.23 – 0.13) | 1.54  (1.19 – 1.89) |
| Q23 | 0.78  (0.67 – 0.86) | 0.39  (0.27 – 0.55) | 0.53  (0.48 – 0.57) | 0.18  (0.04 – 0.32) | 2.23  (1.68 – 2.78) |
| Q29 | 0.68  (0.55 – 0.78) | 0.54  (0.40 – 0.70) | 0.30  (0.26 – 0.34) | -0.51  (-0.71 – -0.31) | 1.50  (1.13 – 1.87) |
| Q34 | 0.74  (0.61 – 0.84) | 0.45  (0.30 – 0.63) | 0.40  (0.36 – 0.44) | -0.20  (-0.36 – -0.04) | 1.65  (1.24 – 2.06) |
| *Model fit: PPP = .19; 95% CI for X^2^ difference = -12.59 – 36.53*  *Cronbach’s Alpha = .77*  *Composite Scale Reliability (95% CI) =0.77 (0.75 – 0.81)*  *Test-retest ICC (95% CI) = 0.91 (0.84 –0 .95)* | | | | | |
|  | **CTT analyses** |  |  | **IRT analyses** |  |
| **Items** | **Factor loading^a^**  **(95% CI^b^)** | **Residual variance^a^**  **(95% CI^b^)** | **Item difficulty (95% CI)** | **Item location (95% CI)** | **Item discrimination (95% CI)** |
| **7. Digital services that suit individual needs** | | | | | |
| Q18 | 0.82  (0.74 – 0.88) | 0.32  (0.22 – 0.45) | 0.60  (0.55 – 0.63) | 0.32  (0.18 – 0.46) | 2.23  (1.82 – 2.64) |
| Q28 | 0.86  (0.81 – 0.91) | 0.26  (0.18 – 0.35) | 0.56  (0.52 – 0.61) | 0.25  (0.13 – 0.37) | 3.44  (2.73 – 4.15) |
| Q31 | 0.86  (0.79 – 0.90) | 0.27  (0.19 – 0.37) | 0.45  (0.41 – 0.49) | 0.00  (-0.12 – 0.12) | 3.15  (2.52 – 3.78) |
| Q33 | 0.90  (0.85 – 0.93) | 0.20  (0.13 – 0.27) | 0.51  (0.46 – 0.55) | 0.05  (-0.11 – 0.21) | 5.56  (4.48 – 6.64) |
| *Model fit: PPP = .24; 95% CI for X^2^ difference = -10.85 – 24.84*  *Cronbach’s Alpha = .88*  *Composite Scale Reliability (95% CI) = 0.88 (0.86 – 0.90)*  *Test-retest ICC (95% CI) = 0.80 (0.63 – 0.89)* | | | | | |
| ^a^Standardized factor loadings and residual variances reported.  ^b^CI^ = credibility interval.  ^c^See truncated items in Multimedia Appendix 2.  CI = confidence interval.  PPP = posterior predictive *P* value.  95% CI for *X^2^* difference = 95% credibility interval for the difference between observed and replicated Chi-square values.  ICC = intraclass correlation coefficient. | | | | | |
