# Supplementary material for: Validity Evidence of the eHealth Literacy Questionnaire (eHLQ) Part 2: Mixed Methods Approach to Evaluate Test Content, Response Process, and Internal Structure in the Australian Community Health Setting
Source: J Med Internet Res. 2022 Mar 8;24(3):e32777. doi: 10.2196/32777 (PMC8941428; doi:10.2196/32777)
Supplement: Multimedia Appendix 4 [file jmir_v24i3e32777_app4.docx]

**Multimedia Appendix 4:** Bayesian model fit information of the eHealth Literacy Questionnaire 7-factor models

| **Model parameter** | **Model 1**  **0.01/300^a^** | **Model 2**  **0.02/300^a^** | **Model 3**  **0.01/200^a^** | **Model 4**  **0.02/200^a^** | **Model 5**  **0.01/150^a^** | **Model 6**  **0.02/150^a^** |
| --- | --- | --- | --- | --- | --- | --- |
| PPP | .08 | .10 | .30 | .35 | **.49** | .52 |
| 95% CI for *X*^2^ difference | -22.65 – 175.15 | -33.772 – 167.42 | -73.86 – 132.24 | -79.60 – 125.38 | **-101.40 – 108.83** | -103.51 – 102.85 |
| PPPP | .66 | .78 | .87 | .90 | **.92** | .93 |
| DIC | 41,740.90 | 41,713.41 | 41,728.15 | 41,701.01 | **41,727.55** | 41,701.59 |
| N iterations to PSR consistently < 1.05 | 5,500 | 6,100 | 3,800 | 6,700 | **3,700** | 4,000 |
| N iterations to PSR consistently < 1.01 | 20,000+ | 20,000+ | 20,000+ | 20,000+ | **10,400** | 19,200 |
| N (%) of significant target loadings | 35 (100%) | 35 (100%) | 35 (100%) | 35 (100%) | **35 (100%)** | 35 (100%) |
| N of sig. cross-loadings | 1 | 0 | 0 | 0 | **0** | 0 |
| N of sig. correlated residuals | 42 | 32 | 50 | 44 | **55** | 46 |
| Range of inter-factor correlations | 0.19 – 0.95 | 0.18 – 0.93 | 0.21 – 0.95 | 0.19 – 0.94 | **0.21 – 0.95** | 0.19 – 0.94 |

^a^Informative priors for cross-loadings/ inverse-Wishart degrees of freedom for residual covariances.

Bold = model fit information of model of interest.

PPP = posterior predictive *P* value.

95% CI for *X*^2^ difference = 95% confidence interval for the difference between observed and replicated Chi-square values.

PPPP = prior-posterior predictive *P* value.

PSR = potential scale reduction.
