# Supplementary material for: Validity Evidence of the eHealth Literacy Questionnaire (eHLQ) Part 2: Mixed Methods Approach to Evaluate Test Content, Response Process, and Internal Structure in the Australian Community Health Setting
Source: J Med Internet Res. 2022 Mar 8;24(3):e32777. doi: 10.2196/32777 (PMC8941428; doi:10.2196/32777)
Supplement: Multimedia Appendix 5 [file jmir_v24i3e32777_app5.docx]

**Multimedia Appendix 5:** Estimates for the direct effect of eHealth Literacy Questionnaire items on administration format, site area and health setting.

| **Scale/Item** | | **Admin format** | **Area** | **Setting** |
| --- | --- | --- | --- | --- |
| **1. Using technology to process health information** | | | | |
| Q7 | I use technology to find… | **0.08 (0.04)** | -0.02 (0.03) | -0.01 (0.04) |
| Q11 | I often use technology… | 0.03 (0.04) | 0.01 (0.03) | -0.02 (0.04) |
| Q13 | Technology helps me… | -0.06 (0.04) | 0.01 (0.04) | -0.02 (0.04) |
| Q20 | I use technology to share… | **-0.08 (0.04)** | 0.02 (0.04) | 0.05 (0.04) |
| Q25 | I use technology to organize… | -0.02 (0.04) | -0.01 (0.04) | 0.02 (0.04) |
| **2. Understanding of health concepts and language** | | | | |
| Q5 | The knowledge I have helps me… | 0.04 (0.04) | 0.07 (0.04) | 0.06 (0.05) |
| Q12 | I have enough information… | 0.05 (0.04) | 0.00 (0.05) | -0.06 (0.05) |
| Q15 | I understand medical results… | 0.00 (0.04) | -0.07 (0.04) | -0.08 (0.05) |
| Q21 | Overall, I understand how… | 0.02 (0.04) | 0.01 (0.05) | 0.00 (0.05) |
| Q26 | I use measurements about… | -0.08 (0.04) | -0.03 (0.04) | 0.04 (0.05) |
| **3. Ability to actively engage with digital services** | | | | |
| Q4 | I know how to use technology… | -0.02 (0.03) | 0.06 (0.03) | 0.01 (0.04) |
| Q6 | I know how to make… | -0.00 (0.03) | -0.01 (0.03) | -0.05 (0.04) |
| Q8 | I can enter data into… | -0.00 (0.03) | -0.02 (0.03) | 0.03 (0.04) |
| Q17 | I quickly learn how to find… | 0.03 (0.03) | -0.03 (0.03) | -0.00 (0.04) |
| Q32 | I easily learn to use new… | -0.02 (0.03) | -0.01 (0.03) | 0.01 (0.04) |
| **4. Feel safe and in control** | | | | |
| Q1 | I am sure that my health data… | 0.04 (0.04) | 0.02 (0.04) | -0.03 (0.05) |
| Q10 | My electronic healthcare data… | 0.06 (0.04) | -0.01 (0.04) | -0.03 (0.05) |
| Q14 | I have a clear understanding… | -0.03 (0.04) | 0.01 (0.04) | 0.05 (0.04) |
| Q22 | I am sure that only authorized… | -0.06 (0.04) | -0.01 (0.04) | -0.02 (0.05) |
| Q30 | I am confident that healthcare… | -0.00 (0.04) | 0.00 (0.04) | 0.03 (0.05) |
| **5. Motivated to engage with digital services** | | | | |
| Q2 | Technology makes me… | 0.07 (0.04) | 0.02 (0.04) | -0.03 (0.04) |
| Q19 | I find technology helps me… | 0.01 (0.04) | -0.03 (0.04) | -0.01 (0.04) |
| Q24 | I find I get better services… | -0.07 (0.04) | 0.03 (0.04) | 0.03 (0.04) |
| Q27 | Technology improves… | 0.00 (0.04) | -0.02 (0.04) | 0.01 (0.04) |
| Q35 | I find technology useful… | -0.02 (0.04) | 0.00 (0.04) | 0.00 (0.04) |
| **6. Access to digital services that work** | | | | |
| Q3 | Information about my health… | **-0.12 (0.04)** | 0.07 (0.04) | -0.04 (0.04) |
| Q9 | My healthcare providers… | **0.09 (0.04)** | -0.07 (0.04) | -0.05 (0.04) |
| Q16 | My health data are available… | **-0.10 (0.04)** | -0.03 (0.04) | -0.05 (0.04) |
| Q23 | All the health technology I use… | 0.02 (0.04) | 0.06 (0.04) | 0.06 (0.04) |
| Q29 | Most of my healthcare… | 0.01 (0.04) | -0.03 (0.04) | 0.06 (0.04) |
| Q34 | I have access to health… | 0.05 (0.04) | 0.01 (0.04) | 0.01 (0.04) |
| **7. Digital services that suit individual needs** | | | | |
| Q18 | I find that eHealth systems… | 0.01 (0.04) | 0.02 (0.04) | -0.05 (0.04) |
| Q28 | I find eHealth systems seem to… | 0.02 (0.04) | 0.02 (0.04) | 0.04 (0.04) |
| Q31 | I find eHealth systems are… | -0.04 (0.04) | -0.02 (0.04) | -0.01 (0.04) |
| Q33 | eHealth systems provide me… | 0.01 (0.04) | -0.02 (0.04) | 0.01 (0.04) |
| Items are truncated. Please contact the authors for full items.  Standardized estimates reported; posterior standard deviation for estimates shown in parentheses.  Bold = statistically significant differences, one-tailed significant if *P* <.025.  Administration format code: 0 = interview, 1 = paper.  Area code: 0 = metropolitan, 1 = regional.  Setting code: 0 = private clinic, 1 = community health. | | | | |
