# Supplementary material for: Validity Evidence of the eHealth Literacy Questionnaire (eHLQ) Part 2: Mixed Methods Approach to Evaluate Test Content, Response Process, and Internal Structure in the Australian Community Health Setting
Source: J Med Internet Res. 2022 Mar 8;24(3):e32777. doi: 10.2196/32777 (PMC8941428; doi:10.2196/32777)
Supplement: Multimedia Appendix 6 [file jmir_v24i3e32777_app6.docx]

**Multimedia Appendix 6:** Item characteristic curves and information function curves of the eHealth Literacy Questionnaire items (Item Response Theory for Patient-Reported Outcomes outputs).

## Scale 1 – Using technology to process health information


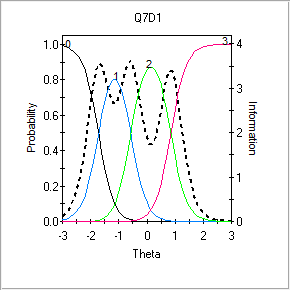

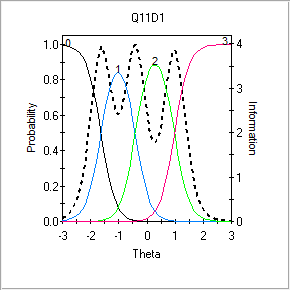


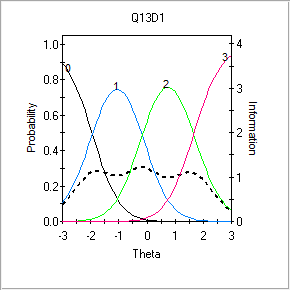

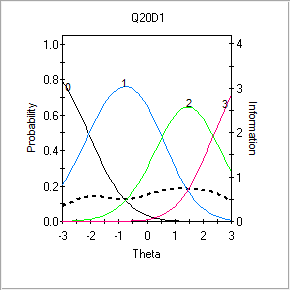


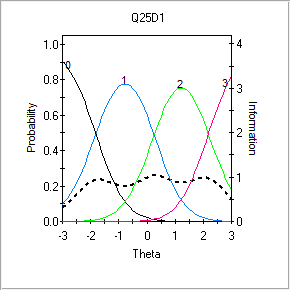


Note: Dotted line = information function curve; 0 = strongly disagree, 1 = disagree, 2 = agree, 3 = strongly agree.

## Scale 2 – Understanding of health concepts and language


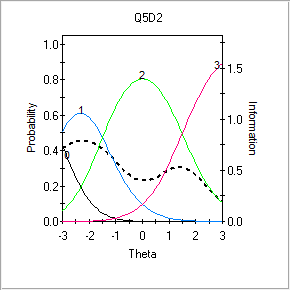

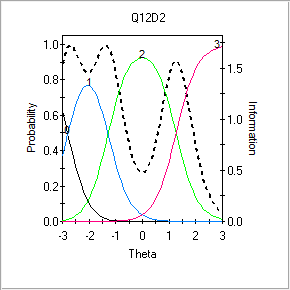


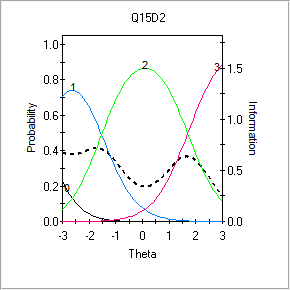

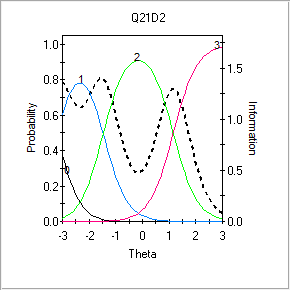


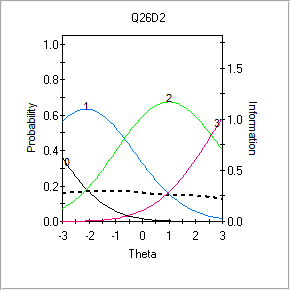


Note: Dotted line = information function curve; 0 = strongly disagree, 1 = disagree, 2 = agree, 3 = strongly agree.

## Scale 3 – Ability to actively engage with digital services


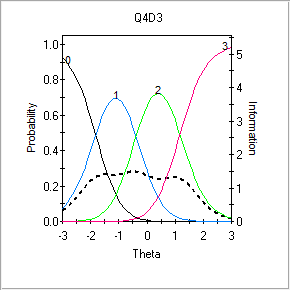

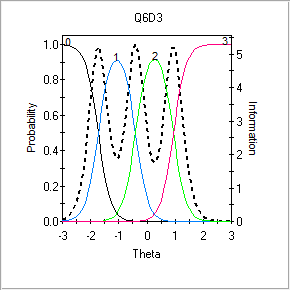


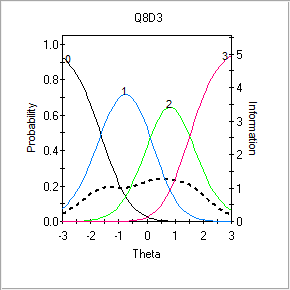

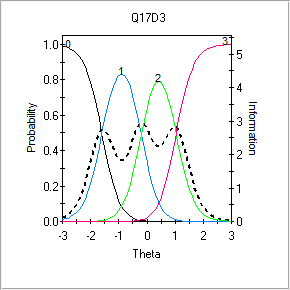


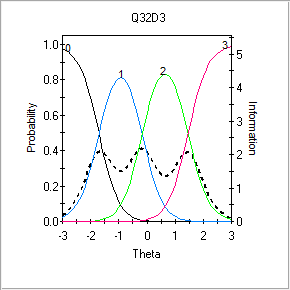


Note: Dotted line = information function curve; 0 = strongly disagree, 1 = disagree, 2 = agree, 3 = strongly agree.

## Scale 4 – Feel safe and in control


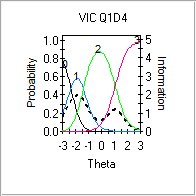

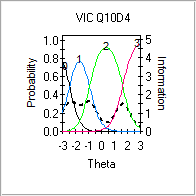


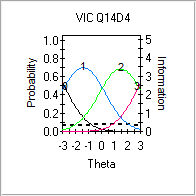

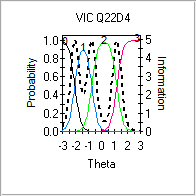


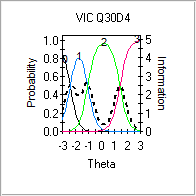


Note: Dotted line = information function curve; 0 = strongly disagree, 1 = disagree, 2 = agree, 3 = strongly agree.

## Scale 5 – Motivated to engage with digital services


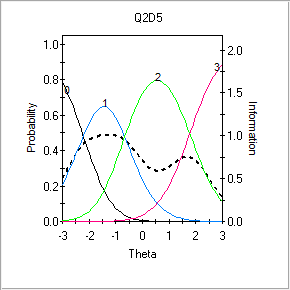

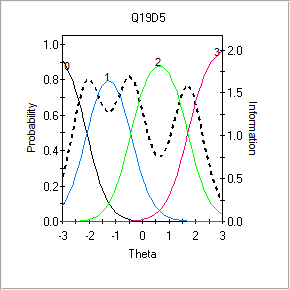


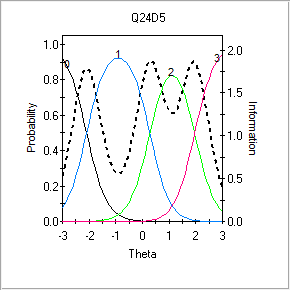

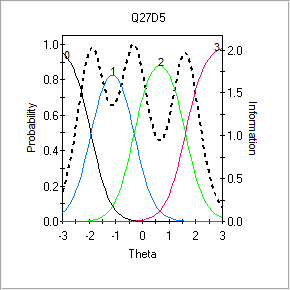


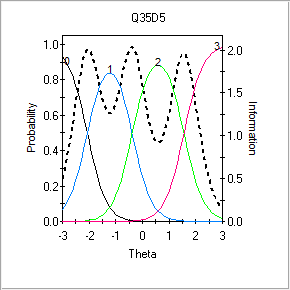


Note: Dotted line = information function curve; 0 = strongly disagree, 1 = disagree, 2 = agree, 3 = strongly agree.

## Scale 6 – Access to digital services that work


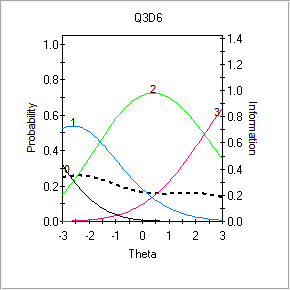

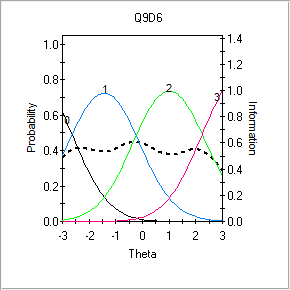


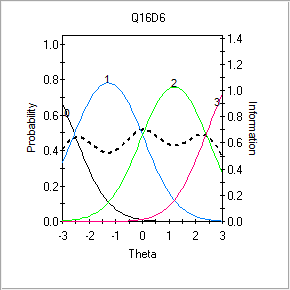

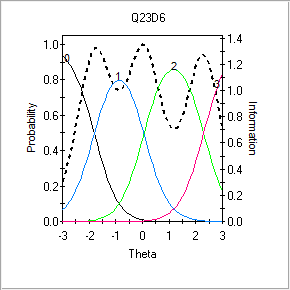


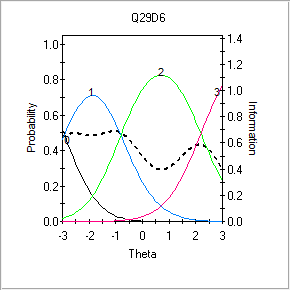

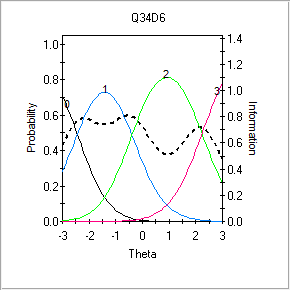


Note: Dotted line = information function curve; 0 = strongly disagree, 1 = disagree, 2 = agree, 3 = strongly agree.

## Scale 7 – Digital services that suit individual needs


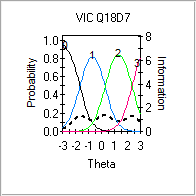

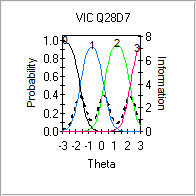


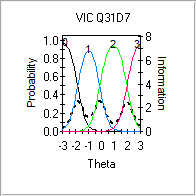

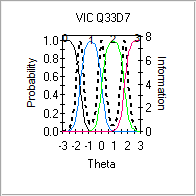


Note: Dotted line = information function curve; 0 = strongly disagree, 1 = disagree, 2 = agree, 3 = strongly agree.
